# Help, near and far: a systematic review of post-COVID digital mental health solutions for domestic violence victims

**DOI:** 10.3389/fpubh.2025.1687396

**Published:** 2026-01-30

**Authors:** Ping Yu, Ping Zhu, Abdulswabul Kudiza, Francis Mungai Kaburu, Mehak Intizar, Chaojun Tong, Ruijie Zhang, Jianlin Jiang, Xin Yu, Qiang Kuang, Ruru Chen, Claudimar Pereira da Veiga, Yu-Tao Xiang, Zhaohui Su

**Affiliations:** 1 School of Public Health, Southeast University, Nanjing, China; 2 Breast Disease Center, Jiangsu Cancer Hospital, Jiangsu Institute of Cancer Research, The Affiliated Cancer Hospital of Nanjing Medical University, Nanjing, China; 3 Fundação Dom Cabral - FDC, Nova Lima, Brazil; 4 Unit of Psychiatry, Department of Public Health and Medicinal Administration, University of Macau, Macao, Macao SAR, China; 5 Institute of Translational Medicine, Faculty of Health Sciences, University of Macau, Macao, Macao SAR, China; 6 Centre for Cognitive and Brain Sciences, Institute of Advanced Studies in Humanities and Social Sciences, University of Macau, Macao, Macao SAR, China; 7 Key Laboratory of Environmental Medicine Engineering, Ministry of Education, School of Public Health, Southeast University, Nanjing, China

**Keywords:** domestic violence, mental health, digital interventions, policy, post-COVID-19 era

## Abstract

**Introduction:**

Domestic violence (DV), as a global pandemic, poses a significant challenge within the field of public health, gravely impacting the mental and physical health of victims. Modern digital technologies have been proposed as promising interventions for DV-related mental health problems. We therefore evaluated their effectiveness.

**Objective:**

To systematically review digital interventions targeting the mental health of DV survivors and to summarize implications for health professionals and policy makers.

**Methods:**

We searched PubMed, EBSCO, and Web of Science (January 1, 2020–April 23, 2024) following PRISMA guidelines. Two reviewers independently screened records, applied predefined eligibility criteria, and extracted data. Owing to heterogeneity, we performed a narrative synthesis. Meanwhile, based on the results of the literature review, this paper proposes a series of policy recommendations from a post-COVID-19 era perspective, integrating societal context and relevant policies.

**Results:**

Nine studies met the inclusion criteria. Three reported no significant mental-health benefits, whereas the remainder showed improvements in outcomes such as depression, anxiety, PTSD symptoms, emotion regulation, perceived support, or safety preparedness using tools including mobile apps, web-based programs, virtual reality, chatbots, and video adjuncts.

**Conclusion:**

Digital interventions show promise for improving mental-health outcomes among DV survivors, but their implementation requires attention to safety, engagement, cultural adaptation, and integration with offline services.

**Systematic review registration:**

PROSPERO, https://www.crd.york.ac.uk/PROSPERO/view/CRD42023488560

## 1 Introduction

Violence against women in domestic settings has been described by the United Nations as a “global pandemic” and a major public health concern (1, 2). The phenomenon of women experiencing domestic violence (DV) is particularly prevalent in low- and middle-income countries, although it also occurs in high-income countries (3, 4). Worldwide, on average, one in three women has experienced DV (5). DV affects women across all age groups, particularly those of reproductive age. In China, 8.6% of married women suffered physical or psychological violence from their spouses. This indicates that the home can be one of the most dangerous settings for women. The adverse effects of DV on health encompass not only physical health but also mental health (6, 7).

Symptoms related to mental health may sometimes remain unexpressed, making them easily overlooked by the public and resulting in a lack of timely intervention and treatment (8). During the COVID-19 pandemic, limitations in identifying mental health needs, coupled with movement restrictions, financial strain, and prolonged cohabitation, heightened survivors' exposure to perpetrators while simultaneously reducing access to in-person psychological, legal, and social services. This context not only amplified existing vulnerabilities but also exposed the limitations of conventional support systems, barriers that have persisted beyond the pandemic. These barriers include stigma, safety concerns, geographical distance, long wait times, and the need to maintain privacy from abusers. As a result, many survivors, particularly those who have experienced long-term abuse or remain “hidden,” continue to be underserved and untreated.

Addressing and treating the mental health issues of DV victims is a significant concern. Recent technological advancements have introduced novel approaches to addressing the mental health issues arising from DV. In high-income countries, technology-based solutions for health problems have been widely implemented in real life. The internet, mobile devices, and smartphones have been utilized in areas such as chronic disease management and mental health challenges (9, 10). The steady increase in internet access rates in low- and middle-income countries in recent years has also laid the groundwork for utilizing relevant technologies to address the mental health issues of DV victims (11). However, research and application of modern information and communication technologies to solve mental health issues for DV victims remain insufficient.

Interventions based on modern digital technology have unique advantages, including: (1) reducing economic costs; (2) breaking geographic barriers, thus providing a broader range of resources to the audience; (3) addressing the accessibility issues of mental health services due to the severe shortage of mental health professionals, such as psychiatrists and counselors, in China. There is a pressing need to introduce digitally mediated psychological treatment methods.

Currently, research on interventions using modern digital technology for DV is still limited (11). Existing literature primarily focuses on screening and identifying DV (12, 13), with a few studies exploring preventive measures (14, 15), while research on how to use modern technology to address the mental health issues of DV victims is even scarcer (16). Moreover, only a limited number of studies have explored decision-making support and assessed the adaptability of modern technology in the context of DV (12). Reported interventions mainly involve tele-counseling, online cognitive behavioral therapy (iCBT), mobile apps for safety planning, AI-based chatbots, and virtual support groups.

In the post-COVID-19 era, the integration of technology and healthcare has become an inevitable trend. Considering (1) the effectiveness and potential economic benefits of mental health interventions based on modern digital technology; (2) the severe negative impacts of DV; and (3) the urgent mental health treatment needs of DV victims, this paper synthesizes existing research on using modern digital technology to address the mental health issues of DV victims and evaluates the effectiveness of various solutions, proposing policy recommendations based on the current policy environment and the results of this evaluation and analysis.

To our knowledge, evidence syntheses specifically evaluating the effectiveness of digital interventions for DV- related mental health outcomes remain limited, particularly in the post-COVID-19 context. This review systematically examines digital mental health interventions for survivors of DV, with the aim of assessing their effectiveness and identifying contextual policy implications for the post-COVID-19 era. This review addresses the following research questions: (1) What types of interventions address the mental health issues of DV victims? What are the specifics of these interventions? Which interventions are effective? (2) What are the causes of violence and abuse faced by DV victims, and what dilemmas do they encounter? (3) Based on the above analysis, what policies can promote the use of modern digital technology to address the mental health issues of DV victims?

## 2 Methods

We followed the Preferred Reporting Items for Systematic Reviews and Meta- Analyses(PRISMA) guidelines to structure the study, details of which can be found in the PRISMA checklist (Appendix 1). The protocol was prospectively registered in PROSPERO (Registration No: CRD42023488560). The population- intervention- comparison- outcome(PICO) framework is shown in Table 1.

**Table 1:** Review framework.

**Population**	**Intervention**	**Comparison**	**Outcome**
Sample size	Intervention plans	No intervention	Suicidal ideation
Age	Duration of intervention	Active control	Depression, anxiety
Gender	Intervention platform		Post-traumatic stress disorder symptoms
			Emotional regulation difficulties
			Perceived support
			Interpersonal sensitivity
			Self-efficacy, etc.

### 2.1 Search strategies

A systematic review was conducted, employing a digital search of bibliographic databases: PubMed, EBSCO, and Web of Science. The literature was systematically screened by titles and abstracts and by applying key search terms. The following search terms were used: DV, intimate partner violence (IPV), family violence; mental health, mental wellbeing, psychological health, psychological wellbeing; digital health, eHealth, mHealth, internet-based intervention, mobile health technologies. Because terminology varies across disciplines and databases, DV was treated as an umbrella term that includes IPV. Accordingly, studies indexed under IPV were included when the abuse was perpetrated by a current or former intimate partner and the recipients were women. The full list of search terms is provided in Appendix 2.

Studies were included if they described an intervention that used some form of Technology-based Tools (TbT), and if the recipients were women who experienced IPV or DV, no matter what was the intervention type, comparison group, outcomes, study design, who was providing the intervention. Only peer-reviewed original studies were included. We excluded studies that did not focus on TbT, studies where interventions were not aimed at women with IPV experiences, studies that described protocols, were not written in English, or were not full text, as well as journal articles and chapters in books. Non-English language articles were excluded because no evidence exists of systematic bias caused by language restrictions (17). The search was limited to studies published between January 1, 2020 and April 23, 2024. Two reviewers independently screened titles/abstracts and full texts, extracted data, and resolved disagreements through discussion applying the Rayyan (developed by Ouzzani et al., etc.) (18) as selection tool. A meta-analysis was not conducted because of the disparities in study designs, variables, and exposures between the studies. The specific inclusion criteria are listed in Table 2.

**Table 2:** Inclusion criteria.

**Category**	**Criteria**
Study population	Women who experienced DV/IPV
Language	English
Research focus	Digital intervention approach and effectiveness
Intervention	Interventions based on modern technology
Study outcome	Reporting the effect of the intervention

## 3 Results

### 3.1 Summary

In total, 1246 articles were identified, among which 247 articles were duplicates. 911 papers were excluded based on the content of their abstracts. The inclusion criteria were then applied to the remaining 88 articles after reading their full text. 79 articles were then excluded, and 9 articles were kept for analysis.

Table 3 lays out the studies in terms of population, intervention, comparison groups. Meanwhile, the authors, publication year, study country, study type, theme and sample size are included. Of the nine included studies, two were conducted in the United States, two in the Netherlands, and the remaining studies were conducted in Kenya, South Africa, the Republic of Korea, Norway, and Canada.

**Table 3:** Key characteristics of included articles.

**Literature**	**Nations**	**Type of study**	**Sample**	**Intervention plan**	**Key outcomes**
Glass et al. (19) (2022)	United States	A randomized controlled trial	*n* = 346 Age: 18–24 female	Intervention: the myPlan interactive decision-aid and safety-planning app; Control: a general safety program website.	Depressive symptoms were reduced; suicide risk was reduced.
Decker et al. (20) (2020)	Kenya	A randomized controlled trial	*n* = 352 Age: 18–35 female	Intervention: the myPlan Kenya app for safe decision-making and planning with support from trained community health volunteers; Control: an app-like web page providing information about intimate partners and resources (legal, health, safety, counseling, and financial).	Safety readiness scores increased significantly; depressive symptoms showed no significant improvement.
van Gelder et al. (21) (2023)	Netherlands	A randomized controlled trial	*n* = 198 Age: 18–50 female	Intervention: the full SAFE eHealth program via a website (information plus interactive components for help and interaction); Control: a basic version with only essential information and available help resources.	Anxiety symptoms were significantly reduced in the intervention group.
van Gelder et al. (22) (2023)	Netherlands	Qualitative research	*n* = 65 Age: 38 (average) female	Intervention: the full SAFE eHealth program (informational support, help database, and ability to contact other DV survivors); Control: a basic version with limited information and help options, without interaction or additional support.	The SAFE eHealth program was perceived as potentially beneficial for providing psychosocial support.
De Filippo et al. (23) (2023)	South Korea	A randomized controlled trial	*n* = 19,643 Age: 18–24 female	Intervention: chatbot-delivered content with three active arms (attention: educational sexual health information; gamification: gamified behavioral economics content; narrative: behavioral economics content via prerecorded voice notes); Control: no intervention.	No significant reduction in depressive symptoms was observed.
Lee et al. (24) (2021)	South Korea	A randomized controlled trial	*n* = 34 Age: 18–24 years female	Intervention: the mobile VR program “Sister, I will tell you!©” featuring a 3D hologram recovery narrative used to guide writing and meditation; Control: audio-guided positive-thinking meditation with the same content, but without VR and personalized storytelling.	Perceived support increased significantly; suicidal ideation decreased significantly.
Flaathen et al. (25) (2022)	Norway	Multicenter randomized controlled trial	*n* = 1,818 Age: over 18 Average age: 31.5 female	Intervention: a tablet-based video including IPV information and help-seeking strategies; Control: videos without IPV-related information.	No significant change in mental health scores was found from pre-intervention to follow-up.
Marilyn Ford-Gilboe et al. (26) (2020)	Canada	Double-blind randomized controlled trial	*n* = 462 Age: over 19	Intervention: the personalized online safety and health program iCAN Plan 4 Safety (interactive activities, hazard assessment with immediate feedback, priority assessment, personalized action plan, and de-emotionalization activities); Control: a non-personalized, static version providing information and access to help resources only.	Depressive symptoms improved significantly; PTSD symptoms improved significantly.
Newlands et al. (27) (2021)	United States	Pilot randomized controlled trial	*n* = 24 Age: 37 (average)	Intervention: a 2-day dialectical behavior therapy (DBT) skills group plus video links sent every other day for 22 days via text/email; Control: the same 2-day DBT skills group only (no video adjunct).	Depressive symptoms decreased significantly; anxiety symptoms decreased significantly; PTSD symptoms decreased significantly; interpersonal sensitivity improved significantly.

### 3.2 Study results

Glass et al. (19) used the myPlan app for an intervention that lasted 12 months, during which time participants were able to update and reassess their safety plans through self-assessment using the app to ensure that the plans were adapted to any new situations or changes in relationships. myPlan apps can provide access to resources for mental health services, which are primarily in the form of links embedded in a personalized safety plan that users can access directly. Intervention results showed that use of the myPlan app reduced suicide risk and improved decision-making skills among victims of DV.

Decker et al. (20) conducted an intervention using the myPlan Kenya app for 3 months, during which users received education on healthy relationships, learned to recognize healthy and unhealthy relationships, assessed for warning signs in the relationship such as extreme jealousy, insults, threats, etc., and were provided with a safety decision-making plan. Results of the intervention showed that use of the myPlan Kenya app did not have a significant impact on reducing levels of depression in victims of DV, but there was a significant increase in safety preparedness (SP) scores.

van Gelder et al. (21) used a website called “SAFE” for an intervention that lasted for 18 months. Through this website, participants had access to information about DV and abuse, information about safety (e.g., how to leave an abusive partner safely), and access to a help database that included type and area filters. The website also provides mental health support and social support, and users can access mental health-related information and resolution strategies. In addition, the website includes an interactive component that allows participants to chat with other victims of DV. Results of the intervention showed that use of the SAFE website reduced anxiety symptoms and increased adjustment in victims of DV.

van Gelder et al. (22) used a website called “SAFE” for their intervention, did not report a specific length of intervention, and the entire program lasted approximately 2 years. Participants were able to access the website to obtain physical and mental health information, social support, use help options, and interact with others. The results of the intervention showed that the qualitative results supported the positive effects of using the website in increasing self-reflection and awareness among victims of DV, and that there were no significant improvements in mental health conditions, such as self-efficacy, depression, and anxiety, among the quantitative results.

De Filippo et al. (23) conducted an intervention through the use of chatbots, a behaviorally informative chatbot, for 3 months. Participants were divided into four groups: a control-only group, an information support therapy group, a gamification intervention therapy group, and a narrative therapy group. Subjects in the information support treatment group were provided with basic information about sexual health through the chatbots in text form, and were also able to navigate through different topic areas; users in the gamification intervention treatment group were able to interact with the bots to learn how to identify and respond to unhealthy relationship behaviors, healthy communication and coping mechanisms, and to develop a safety plan, while receiving a symbolic reward; and the narrative The treatment group receives an intervention in the form of a narrative through pre-recorded voice notes and short textbooks, in which the user role-plays and emotionally engages to understand and cope with problems in relationships. The results of the intervention showed that the use of chatbots in a slight but not statistically significant improvement on depressive symptoms.

Lee et al. (24) conducted mobile therapy through virtual reality technology with a program called “Sister, I will tell you!©” that lasted 4 weeks. Participants received a mobile virtual intervention for reflective writing and positive thinking meditation. Using a smartphone with a reflective pyramid device, they were able to see a 3D holographic image of a female character who shared her healing story and guided reflective writing and positive thinking meditation. The character, who is approximately the same age as the participant, facilitates empathy and engagement by recounting her experiences. This setup helps users visually immerse themselves in the virtual environment to enhance the appeal and interactivity of the intervention. The results of the intervention show that treatment with this technology enhances perceived support and attenuates Suicidal Ideation in victims of DV.

Flaathen et al. (25) delivered the intervention through a video, which was played on a tablet computer and was 7 min long; the researchers did not report the total length of time spent receiving the intervention. The videos contained information about DV and safe behaviors. The study was assessed using the World Health Organization Quality of Life-Brief (WHOQOL-BREF). The results of the study showed that receiving the intervention did not significantly improve the psychological status of victims of DV.

Ford-Gilboe et al. (26) conducted an intervention through a customized, interactive online tool called “i CAN Plan 4 Safety” over a 12-month period. Participants were able to access a variety of resources and support during the intervention, including personalized safety planning and health information, a hazard assessment tool, and an action plan based on user responses. Findings showed that the program was able to improve victims of DV's depressive symptoms, Post-Traumatic Stress Disorder (PTSD) symptoms, and increase their perception of social support and control.

Newlands et al. (27) conducted an intervention through a video-based approach to intervention (Video Intervention Adjuncts, VIA) for 22 days. Participants took part in 2 days of Dialectical Behavior Therapy skills, including positive thinking, emotion regulation, dilemma tolerance, identification, and interpersonal interactions. Upon completion, depending on the experimental condition, participants received a video intervention add-on via text message or email every other day for review of core skills. Video content covered wise mindfulness, behavioral chaining, fact-checking, opposite action, interpersonal effectiveness, distress tolerance, radical acceptance, validation/clarity, invalidation, and self-validation. The results of the study showed that the intervention had a significant positive effect on improving emotion regulation, depressive symptoms, anxiety, interpersonal sensitivity, and PTSD symptoms in victims of DV.

### 3.3 Characteristics of female participants in the included studies

Across the included studies, the majority of participants were women with a confirmed history of domestic or IPV. Although demographic characteristics varied across countries, several common patterns emerged. Most participants were adults of reproductive age who exhibited symptoms of depression, anxiety, or PTSD, and a considerable proportion lacked stable social support. In studies conducted in low- and middle-income settings, financial dependence on partners and limited access to professional counseling were frequently reported.

### 3.4 Effectiveness evaluation

Common psychological problems among victims of DV include (PTSD), depression, anxiety, loss of self-esteem and self-worth, isolation and socialization disorders, and dysfunction (28). This pattern aligns with large-scale epidemiological findings in DV research, which consistently identify depression and anxiety as the most prevalent mental health outcomes among survivors. For the purposes of this review, qualitative studies were considered “effective” when the original authors reported perceived usefulness or acceptability. For randomized controlled trials (RCTs), an intervention was considered “effective” if, at *p* < 0.05, at least one prespecified mental health outcome differed significantly between the intervention and comparator groups at post-intervention (or follow-up). Where sufficient data were available, effect sizes were calculated using Cohen's d (Equation 1) based on post-intervention (or follow-up) group means (*M*_1_ and *M*_2_ for the intervention and comparator groups, respectively) and the pooled standard deviation (*SD*_pooled_; Equation 2). In addition, we extracted and reported pre–post changes in outcome values within the intervention group to describe absolute changes over time. When relevant data were insufficient, effect size calculation was not possible. The specific results are shown in Table 4.


$$\begin{array}{l} d=\frac{M_{1}-M_{2}}{SD_{pooled}} \end{array}$$ (1)

$$\begin{array}{l} SD_{pooled=}\sqrt{\frac{\left(n_{1}-1\right)SD_{1}^{2}+\left(n_{2}-1\right)SD_{2}^{2}}{n_{1}+n_{2}-2}} \end{array}$$ (2)

**Table 4:** Effectiveness evaluation.

**Literature**	**Effectiveness**	**Effect size**
Glass et al. (19 ) (2022)	Depression score: decrease 4.35 (31.04 → 26.69) ; mean suicide risk: decrease 0.1 (0.46 → 0.36)	/
Decker et al. (20) (2020)	Safety readiness score: increase 1.53 (34.52 → 36.05)	*d* = 0.24, significant but small
van Gelder et al. (21) (2023)	Anxiety score: decrease 1.25 (12.80 → 11.55)	/
van Gelder et al. (22) (2023)	Qualitative research	/
De Filippo et al. (23) (2023)	No statistically significant improvement reported	
Lee et al. (24) (2021)	Perceived support: increase 5.4 (23.2 → 28.6); suicidal ideation: decrease 6.8 (14.1 → 7.3)	/
Flaathen et al. (25) (2022)	No statistically significant improvement reported	
Marilyn Ford-Gilboe et al. (26) (2020)	Depression score: decrease 10.97 (39.89 → 28.92); PTSD score: decrease 7.90 (52.35 → 44.45)	/
Newlands et al. (27) (2021)	Depression score: decrease 10.59 (53.63 → 43.04); anxiety score: decrease 9.46 (53.00 → 43.54); difficulty in emotional regulation score: decrease 26.92 (98.00 → 71.80) interpersonal Sensitivity Score: decrease 4.83 (51.33 → 46.50); PTSD score: decrease 19.88 (49.83 → 29.95)	Depressive symptom: *d* = 1.09, significant and large effects; Anxiety symptoms: *d* = 0.80, significant and large effects; Interpersonal sensitivity: *d* = 0.48 Medium effects; PTSD symptoms: *d* = 1.15, significant and large effects; Emotional regulation: *d* = 1.10, significant and large effects

PTSD, post-traumatic stress disorder; d, Cohen's d (effect size).

“baseline → post-intervention” indicates within-intervention-group change over time.

Cohen's d was calculated using post-intervention (or follow-up) group means for the intervention and comparator groups and the pooled standard deviation; effect sizes were reported only when sufficient data were available.

## 4 Discussion

### 4.1 Current situation, constraints and challenges

The overall effectiveness reported across studies was positive but varied. Digital interventions showed stronger improvements in depression, anxiety, and coping when combined with professional guidance or follow-up, while self-directed tools had weaker or non-significant effects. These differences suggest that outcomes depend on user engagement, continuity of support, and access to psychological services. Based on these findings, the realities experienced by survivors—such as financial dependence, cultural norms, and limited access to offline care—should be carefully considered when exploring the implementation of digital interventions in China.

In low- and middle-income countries, DV is driven by deep-seated gender inequality, limited communication skills among women, and insufficient social support (23). Cultural norms often place women in subservient roles within families, leading abusers to feel justified in their actions without fear of social or legal consequences. Women, often unable to access adequate support, endure violence in silence due to prevailing cultural beliefs about “family privacy”.

Economic factors also play a role (26). With traditional family structures designating men as breadwinners and women as homemakers, women struggle to gain financial independence, reinforcing male authority and control within households. Meanwhile, when it comes to escaping abuse, DV victims face both internal and external obstacles in leaving abusive situations (29). Internally, many suffer from low self-esteem and emotional regulation issues (27), often rooted in prolonged abuse, which makes it difficult for them to seek help. Externally, cultural norms that reinforce male dominance and economic dependency, along with legal and family-related constraints, can trap victims in abusive relationships (20–22), shame and fear further inhibit victims' willingness to report abuse (21, 22).

Victims in rural areas often have even fewer resources, facing limited access to support services (26), while young victims may lack professional assistance entirely (19). Digital tools can offer support, yet these often require guidance and access that victims may not have (27). While digital mental health interventions present potential avenues of support, they also come with security and privacy risks. Abusers can exploit victims' devices to monitor or control them (19–22, 29).

The effectiveness of many digital tools is still under review, and technical, financial, and staffing limitations restrict access to these tools (27). For digital interventions to be effective, they must be culturally relevant and customized to address individual needs (19, 20), taking into account specific environmental factors. Building upon these findings, it is essential to consider how digital interventions can be supported through legal and policy frameworks.

### 4.2 Policy analysis

China has gradually established a legal and policy framework to address DV, including the Anti-Domestic Violence Law, the Women's Development Outline, and the Protection Order system. Rather than cataloging every policy detail, it is more important to highlight how these mechanisms can underpin digital psychological interventions. For example, the Protection Order system provides a legal basis for remote documentation of abuse and online evidence submission, which can be integrated into safety-planning apps. Public service hotlines such as 12388 and 110 provide official crisis referral channels that digital platforms could link to. In addition, government support for internet-based health services provides opportunities for online counseling, virtual peer support, and tele-psychology.

However, policy presence alone does not guarantee impact. Awareness of reporting options and protection orders remains low, especially in rural areas, and digital access may be limited among older women. Therefore, while policy frameworks create enabling conditions, digital tools must provide clear guidance, privacy protection, and direct referral pathways in order to be practically useful. Based on the results of this study, the applicability of digital psychological interventions in China is supported by several contextual and demographic factors. Digital psychological interventions show clear feasibility in China due to both user characteristics and technological conditions. The demographic profiles of women in the included studies–mainly adults of reproductive age with depression, anxiety, PTSD symptoms, and limited social support–closely match the profiles of Chinese survivors identified in national surveys. This similarity suggests that the intervention formats and therapeutic mechanisms tested internationally are likely to be applicable in the Chinese context.

China also has one of the world's highest rates of smartphone ownership and internet accessibility (34). Most women, including those in low-income or remote areas, use mobile communication tools in daily life. As a result, digital interventions such as mobile apps, online counseling, anonymous peer-support communities, and virtual reality-based therapy can be delivered through platforms that users already rely on. In rural communities, where mental health professionals and shelters are limited, digital services can reduce geographical and financial barriers.

A post-COVID-19 perspective is used as a starting point to analyze policies relevant to applying modern digital technologies to address DV-related mental health problems, as detailed in Table 5.

**Table 5:** Evidence-based policy recommendations.

**Research finding**	**Policy**	**Theory**
Internet-delivered video interventions can improve mental health outcomes among DV victims.	Strengthening legal support and adding online remote service system	
Effectiveness of scientific and technological interventions; the dilemma of insufficient resources	Build a global network of cooperation to discover potential resource	
Application-based communication support, listening to and reflecting on the stories of avatars can be effective in improving the mental health of DV victims	Establishment of a virtual community to provide peer support	Peer education theory (24): a way of communication and a way of dealing with problems, in which peer educators carry out various service activities to induce the educated to take practical action to change their own behavior, body awareness, attitudes or behaviors
Effectiveness of scientific and technological interventions; the dilemma of insufficient specialized personnel	Establishment of a specialized health care system to train professionals	

#### 4.2.1 Legal support and remote service system development

Digital tools can help bridge these gaps by offering confidential and remote access to legal information and emergency support. Mobile applications and online platforms can guide users in documenting abuse, applying for a personal protection order, and connecting with helplines. Features such as quick-exit features, disguised interfaces, and encrypted communication can enhance user safety when perpetrators monitor devices. Online legal consultation, virtual case reporting, tele-psychology (32), and anonymous peer support can be delivered through systems that Chinese women already use, including mobile apps, messaging platforms, and online communities.

To be effective, digital systems should be integrated with existing offline services. When users express urgent risk, platforms should enable direct referral to police hotlines (110), Women's Federation services (12,338), shelters, hospitals, or community service centers (33, 35). Local governments and NGOs can also develop remote safety- planning programs, online reporting portals, and real-time emergency response mechanisms. In the long term, a coordinated “online and offline” system—linking psychological support, legal assistance, and crisis intervention—may provide a safer and more sustainable pathway for survivors in different living conditions.

#### 4.2.2 Building virtual communities for peer support

China has piloted virtual communities for AIDS patients, demonstrating that shared experiences can significantly benefit users' mental health (24). Listening to stories or connecting with other DV survivors through virtual platforms has shown to improve victims' well-being. Research highlights that peer education can be an effective means to promote change, especially when the information comes from individuals with shared experiences (30, 31).

A virtual community for DV victims could offer a post-pandemic solution, with modules for story-sharing, peer support groups, and resource links to provide counseling and legal aid. The platform should allow users to connect with others based on demographics or experiences, with anonymous communication options available (36). Collaboration with government and non-governmental organizations can enhance access to professional resources.

#### 4.2.3 Establishing specialized medical systems and training

The development of digital intervention systems faces challenges of limited funding and a shortage of trained professionals. The “14th Five-Year Plan” emphasizes the need for specialized healthcare services for women, integrating psychological and preventive health care (29). Hospitals and related institutions should build specialized teams and offer both online and offline services for victims. Key actions include increased funding for women's health specialties, developing specialized courses in partnership with experts in DV-related mental health, and forming multidisciplinary teams of therapists and social workers to provide rapid and ongoing support.

## 5 Conclusion

This review demonstrates that digital mental health interventions offer a promising pathway for supporting survivors of DV, particularly in post-COVID contexts where traditional services may be inaccessible or unsafe. Technologies such as mobile applications, web-based platforms, virtual reality, and chatbot-assisted therapy can improve safety behaviors, emotional regulation, depressive symptoms, anxiety, and PTSD in certain populations. However, the evidence remains fragmented, with variation in study design, follow-up length, and outcome measures.

In China, sociocultural stigma, economic dependence, limited mental health resources, and geographic disparities continue to create substantial barriers to help-seeking. Digital solutions could help address these obstacles, but careful localization, privacy protection, and integration with existing legal and social support systems are essential. Future research should adopt larger samples, longer follow-ups, and mixed-method designs to better evaluate effectiveness and user experience. Policymakers and service providers may consider combining digital tools with community-based services to build a comprehensive, accessible, and survivor-centered support network.

## Data Availability

The original contributions presented in the study are included in the article/Supplementary material, further inquiries can be directed to the corresponding author/s.
