# Supplementary material for: Help, near and far: a systematic review of post-COVID digital mental health solutions for domestic violence victims
Source: Front Public Health. 2026 Jan 30;13:1687396. doi: 10.3389/fpubh.2025.1687396 (PMC12903268; doi:10.3389/fpubh.2025.1687396)
Supplement: Supplementary file 2 [file Presentation_2.pdf]

# Appendix 1 Search Term

**PubMed**

（"domestic violence" [MeSH Terms] OR "intimate partner violence" [MeSH Terms] OR "family violence" [Text Word] OR "spousal violence" [Text Word] OR "violence against women" [Text Word] OR abuse* [Text Word] OR batter* [Text Word] OR violen* [Text Word]）

AND （"mental health" [MeSH Terms] OR "psychological well-being" [MeSH Terms] OR "mental wellbeing" [TIAB] OR "psychological wellbeing" [TIAB] OR "psychological health" [TIAB] OR "mental well-being" [TIAB] OR "psychotherapy" [MeSH Terms] OR psychotherapy [Text Word] OR intervention [Text Word] OR treatment [Text Word]）

AND （"telemedicine" [MeSH Terms] OR "telepsychiatry" [Text Word] OR "telehealth" [Text Word] OR "telecare" [Text Word] OR "telemental health" [Text Word] OR "eHealth" [Text Word] OR "electronic health" [Text Word] OR "uHealth" [Text Word] OR "ubiquitous health" [Text Word] OR "mHealth" [Text Word] OR "mobile health" [Text Word] OR "connected Health" [Text Word] OR "online intervention" [Text Word] OR "internet-based intervention" [Text Word] OR "internet health" [Text Word] OR "internet technology" [Text Word] OR "web-based intervention" [Text Word] OR "social media" [Text Word] OR "social network" [Text Word] OR "Facebook" [Text Word] OR "Twitter" [Text Word] OR "Weibo" [Text Word] OR "microblog" [Text Word] OR "WeChat" [Text Word] OR "mobile technology" [Text Word] OR "mobile devices" [Text Word] OR "mobile health technologies" [Text Word] OR "mobile phone" [Text Word] OR "cell phone" [Text Word] OR "smartphone" [Text Word] OR "smartphone application" [Text Word] OR "smartphone app" [Text Word] OR "smartphone technology" [Text Word] OR "app" [Text Word] OR "mobile app" [Text Word] OR "text message" [Text Word] OR "SMS" [Text Word] OR "short message service" [Text Word] OR "text messaging" [Text Word] OR "artificial intelligence" [Text Word] OR "digital health" [Text Word] OR "digital medicine system" [Text Word] OR "wearable device" [Text Word] OR "virtual reality"）

Filter：Clinical Trial, Randomized Controlled Trial

**Web of Science**

TS=（"domestic violence" OR "intimate partner violence" OR "family violence" OR "spousal violence" OR "violence against women" OR abuse* OR batter* OR violen*）

AND TS=（"mental health" OR "psychological well-being" OR "mental wellbeing" OR "psychological wellbeing" OR "psychological health" OR "mental well-being" OR psychotherapy OR intervention OR treatment）

AND TS=（"telemedicine" OR "telepsychiatry" OR "telehealth" OR "telecare" OR "telemental health" OR "eHealth" OR "digital health" OR "mHealth" OR "mobile health" OR "online intervention" OR "internet-based intervention" OR "web-based intervention" OR "social media" OR "Facebook" OR "Twitter" OR "Weibo" OR "WeChat" OR "mobile technology" OR "smartphone app" OR "app" OR "text messaging" OR "SMS" OR "artificial intelligence" OR "digital therapy" OR "virtual reality" OR "machine learning" OR "digital mental health" OR "health tech" OR "e-mental health"）

Filter：Clinical Trial

**EBSCO**

（TI （"domestic violence" OR "intimate partner violence" OR "family violence" OR "spousal violence" OR "violence against women"） OR AB （abuse* OR batter* OR violen*））

AND （TI （"mental health" OR "psychological well-being" OR "mental wellbeing" OR "psychological wellbeing" OR "psychological health" OR "mental well-being"） OR MM "Psychotherapy" OR TX （psychotherapy OR intervention OR treatment））

AND （TI （"telemedicine" OR "telepsychiatry" OR "telehealth" OR "telecare" OR "telemental health" OR "eHealth" OR "electronic health" OR "uHealth" OR "ubiquitous health" OR "mHealth" OR "mobile health" OR "connected Health" OR "online intervention" OR "internet-based intervention" OR "internet health" OR "internet technology" OR "web-based intervention" OR "social media" OR "social network" OR "Facebook" OR "Twitter" OR "Weibo" OR "microblog" OR "WeChat" OR "mobile technology" OR "mobile devices" OR "mobile health technologies" OR "mobile

phone" OR "cell phone" OR "smartphone" OR "smartphone application" OR "smartphone app" OR "smartphone technology" OR "app" OR "mobile app" OR "text message" OR "SMS" OR "short message service" OR "text messaging" OR "artificial intelligence" OR "digital health" OR "digital medicine system" OR "wearable device" OR "virtual reality" OR "virtual reality intervention" OR "machine learning" OR "computer-assisted therapy"））
